# Retraction: Isoflurane Preconditioning Confers Cardioprotection by Activation of ALDH2

**DOI:** 10.1371/journal.pone.0292458

**Published:** 2023-09-28

**Authors:** The PLOS ONE Editors

Following the publication of this article [1], concerns were raised regarding Figures 1–5. Specifically,

- There appear to be irregularities in the reporting of the graphs presented in Figures 1–5:
  - The error bars on all bar charts are shaped irregularly, despite the reported use of SPSS software in the study’s methodology.
  - The axes on all bar charts are shaped irregularly, despite the reported use of SPSS software in the study’s methodology.
- The following panels appear to partially overlap, despite being used to represent different experimental conditions:
  - Figure 3B: Control RFP panels Ad-Si-ALDH2-RFP, Ad-RFP+Isoflurane, and Ad-Si-ALDH2-RFP + Isoflurane
  - Figure 3B: Control TUNEL panels Ad-Si-ALDH2-RFP, Ad-RFP+Isoflurane, and Ad-Si-ALDH2-RFP + Isoflurane
  - Figure 4B: Control RFP panel Ad-CA-ALDH2-RFP, and H/R RFP panels Ad-CA-ALDH2-RFP and Ad-RFP + Isoflurane
  - Figure 4B: Control RFP panels Ad-RFP + Isoflurane and Ad-CA-ALDH2 RFP + Isoflurane
  - Figure 4B: Control TUNEL panel Ad-CA-ALDH2-RFP, and H/R TUNEL panels Ad-CA-ALDH2-RFP and Ad-RFP + Isoflurane
  - Figure 4B: Control TUNEL panels Ad-RFP + Isoflurane and Ad-CA-ALDH2 RFP + Isoflurane

The first author stated that the concerns raised regarding panel overlaps between Figure 3B and Figure 4B were due to errors made during preparation of the figures and were therefore misrepresented in the published article. The *PLOS ONE* Editors remain concerned about the number of panels affected by figure preparation errors. The authors did not provide a response to the concerns regarding error bars and axes in Figures 1–5.

The original data underlying the published results have not been provided for editorial review. In the absence of original data, these issues cannot be resolved.

In light of the concerns affecting multiple figure panels that question the reliability and integrity of these data, the *PLOS ONE* Editors retract this article.

All authors either did not respond directly or could not be reached.
